# Prevalence and Associated Factors of Anemia among Breast Cancer Patients Undergoing Chemotherapy: A Prospective Study

**DOI:** 10.1155/2022/7611733

**Published:** 2022-04-14

**Authors:** Fares M. S Muthanna, Mahmathi Karuppannan, Egbal Abdulrahman, Suriyon Uitrakul, Bassam Abdul Hassan Rasool, Ali Haider Mohammed

**Affiliations:** ^1^Department of Pharmaceutical Care, School of Pharmacy, Walailak University, Nakhon Si Thammarat 80160, Thailand; ^2^Drug and Cosmetic Excellence Center, Walailak University, Nakhon Si Thammarat 80160, Thailand; ^3^Department of Pharmacy Practice, Universiti Teknologi MARA Cawangan Selangor, Puncak Alam Campus, Bandar Puncak Alam, 42300 Selangor, Malaysia; ^4^Department of Clinical Pharmacy and Pharmacy Practice, University of Science and Technology, Sana'a, Yemen; ^5^Department of Pharmacy, Al Rafidain University College, Baghdad 10001, Iraq; ^6^School of Pharmacy, Monash University Malaysia, Jalan Lagoon Selatan, Bandar Sunway, Kuala Lumpur 47500, Selangor, Malaysia

## Abstract

**Purpose:**

The purpose of this study was to ascertain the prevalence and factors associated with anemia (hemoglobin [Hb] 12 g/dL) in breast cancer patients undergoing chemotherapy.

**Materials and Methods:**

We conducted a prospective longitudinal study to collect demographic and clinical data on adult breast cancer patients with or without anemia who were admitted to HKL, UMMC, and NCI. The incidence of anemia was determined by detecting whether or not anemia developed during the course of chemotherapy. Mild, moderate, or severe anemia was defined. A chi-squared and logistic regression model were used to assess the effect of demographic and clinical factors on the incidence of anemia and multiple logistic regression analysis was used to evaluate the associations of potential risk factors with the presence of CRA.

**Results:**

The study enrolled a total of 292 breast cancer patients. Anemia occurred at a rate of 41.1% (*n* = 120). Our findings indicated that clinical factors such as the number of chemotherapy regimens, dose reduction, and type of chemotherapy, for example, docetaxel, as well as demographic covariates such as age and BMI, all contribute to the incidence of anemia in cancer patients.

**Conclusions:**

According to this study, the prevalence of anemia in breast cancer patients is high. Patients' age, BMI, number of chemotherapy regimens, and docetaxel were risk factors; thus, protocols are needed to identify subgroups of breast cancer likely to benefit from novel management strategies.

## 1. Introduction

Anemia is common in cancer patients, with incidence rates ranging from 22.7% [1] to 63% [2] and increasing to 89% following chemotherapy [3]. Increase incidence of anemia has been associated with reduced treatment response and quality of life of breast cancer patients [4, 5] and raises the financial burden of the disease [6]. Specific subgroups of patients are more likely to develop anemia during the course of the disease, according to Seshadri et al. [7] in a 6-month follow-up survey of cancer patients. Researchers concluded that 73% of patients receiving a combination of both chemotherapy and radiation had anemia, compared to only 58% of those receiving chemotherapy alone. Additionally, the successfully completed European Cancer Anemia Survey (ECAS), a huge multinational prospective survey, provided data on the incidence, and clinical characteristics (e.g., disease status, tumor type, and treatment status) of anemia in a diverse population of patients [8]. Anemia was found to be associated with poor performance status in general; a hemoglobin (Hb) level of 9.7 g/dL was used to initiate anemia treatment.

The prevalence of anemia in cancer patients is well documented. According to Xu et al. [3], patients with anemia who are hospitalized to oncology units frequently report feeling more fatigued and experiencing higher mortality rates, as well as developing more comorbidities than those who are not diagnosed with anemia [5].Thus, knowing the prevalence rates of anemia will aid in determining which groups are most likely to benefit from treatment. Numerous obstacles exist when attempting to describe the prevalence of anemia in cancer patients. First, a large sample size must be amassed in order to assess the various types of cancer patients. Second, it is critical to take into account the risk of mortality in a population that is already very sick and suffering from cancer.

Several factors are associated with the prevalence of anemia in cancer patients. The most common factors include demographic factors [9], biological factors [10], chemotherapy [11], and types of cancer [9]. Socio-demographic factors including increasing age [9, 12]; race, for example, Hispanics [9]; and gender, for example, women, played a significant role in the incidence of anemia in cancer patients. In addition, number of chemotherapy regimens [13, 14], type of chemotherapy [9, 13], and chemotherapy dose delay or dose reduction [15] all increased incidence of cancer related anemia (CRA). Moreover, cancer itself [13, 16] and type of cancer [16, 17] are associated significantly with anemia among cancer patients. Furthermore, the impact of cancer (by direct invasion of bone marrow), cancer treatment (surgery, hormonal therapy, radiotherapy, and targeted therapy), and the effect of cytokines released by cancer cells all contributed to increases prevalence of anemia in cancer patients [18].

The purpose of this study was to examine the prevalence of and factors contributing to cancer-related anemia in a large sample of breast cancer patients admitted to three oncology centers in Malaysia and to provide a basis for future management.

## 2. Methods

After obtaining approval from the Ministry of Research and Ethical Centre (MREC) and institutional review board, we conducted a prospective longitudinal and observational multicenter study of adult patients (aged 18 years or older) admitted to the oncology departments and oncology daycares of Hospital Kuala Lumpur (HKL), University of Malaya Medical Centre (UMMC), and National cancer Institute (NCI) between July 1, 2019, and March 31, 2020. All patients were followed up three times starting from cycle II. Patients with Hb levels of ≤12 g/dL (during all follow-ups) or those diagnosed with breast cancer and undergoing chemotherapy alone were included, while patients receiving other types of treatment, such as radiotherapy, surgery, or hormonal therapy, or those in their first cycle, and those suffering from mental or cognitive disorders and unwilling to participate, were excluded.

### 2.1. Sample Size Determination

The sample size was calculated using the single population proportion formula and based on the incidence of anemia, which ranged from 10 to 40%, assuming *p* = 0.5 and *n* = 384. Since the number of patients during the data collection period is less than 10,000, a correction formula is used. NF = *n*/1 + *n*/N = 384/1 + 384/750 = 253, where NF  is desired sample size, *n* is calculated sample size, and N denotes the total population. After adjusting for a 10% nonresponse rate, the final sample size was 292.

### 2.2. Sampling Techniques

Data were collected from the three major hospitals. First, Hospital Kuala Lumpur (HKL) is a large government hospital in Malaysia that serves as a tertiary and referral hospital in Kuala Lumpur. It has approximately 11300 employees and 2300 beds. Second, the University of Malay Medical Centre (UMMC) in Kuala Lumpur has approximately 1617 beds and serves as a teaching hospital for the University of Malaya. Third, the National Cancer Institute (NCI) is a government cancer treatment facility in Putrajaya, Selangor. It has approximately 252 beds and was specifically designed to provide specialized facilities for cancer patients.

The oncology units and daycare departments of those patients who met the inclusion criteria were identified using their medical records. A one-year review of adults receiving chemotherapy cancer treatment revealed more than 8,000 cases in the oncology units of the three cancer centers.

### 2.3. Study Outcomes

Hb levels were determined in patients' medical records based on blood analysis, and patients with Hb levels ≤12 g/dL (during all follow-ups) were defined as anemic breast cancer patients. Hemoglobin levels ≥12 g/dL were considered normal (anemia not present). According to the National Cancer Institute, anemia is classified as mild (10 g/dL ≤ Hb - 12 g/dL), moderate (8 g/dL ≤ Hb - 10 g/dL), or severe (Hb ≤ 8 g/dL) [19]. We determined the prevalence of anemia at the third follow-up using the same cutoff points as we did at the first and second follow-ups. Patients whose Hb level increased by more than 12 g/dL at any subsequent follow-ups were classified as nonanemic.

### 2.4. Data Collection

Demographic data (e.g., age, ethnicity, marital status, and BMI) were collected and analyzed at all follow-ups. Marital status was classified as married, single, and divorced. Race was categorized as Malay, Indian, Chinese, and others. Patients were classified according to their BMI as obese (BMI kg/m^2^ ≥ 25) or nonobese (BMI kg/m^2^ < 25).

Clinical data (e.g., cancer stage, chemotherapy dose delay, dose reduction, type, and number of chemotherapy) were collected and analyzed at all follow-ups. Cancer stages were divided into four categories: Stage I, Stage II, Stage III, and Stage IV. The number of chemotherapy regimens was divided into two categories: one regimen and more than one regimen. Dose delay is defined as a chemotherapy dose that is delayed for more than 7 days [20], and dose reduction is defined as a dose reduction of at least 10% at any consecutive cycle [21, 22].

### 2.5. Statistical Analysis

Data were analyzed using SPSS version 23. We used regression analysis to examine the relationship between continuous variables and study outcomes, and chi-squared tests to examine the relationship between categorical and ordinal variables. We looked at how factors (e.g., age and race) affected the relationships between independent variables and outcome variables.

Continuous variables were analyzed using the descriptive statistics (mean and standard deviation (SD)). Univariate analyses were conducted using the chi-squared or Fisher exact tests, whereas parametric analyses were conducted using logistic regression analysis. Three steps were taken in analyzing the risk factors for anemia. First, we examined the relationship between potential anemia risk factors and observed anemia (anemic/nonanemic) using univariate analysis for categorical data. Second, all variables with a *p*-value < 0.05 significance level in univariate analysis (chi-squared or Fisher exact tests) were entered into a stepwise logistic regression model. Third, we examined only risk factors with a statistically significant *p*-value. The adjusted odds ratio, 95% confidence intervals (95% CI), and *p*-value are all included in the results of logistic regression analyses. The level of significance was set at a *p*-value < 0.05.

### 2.6. Data Extraction

**Figure 1:**
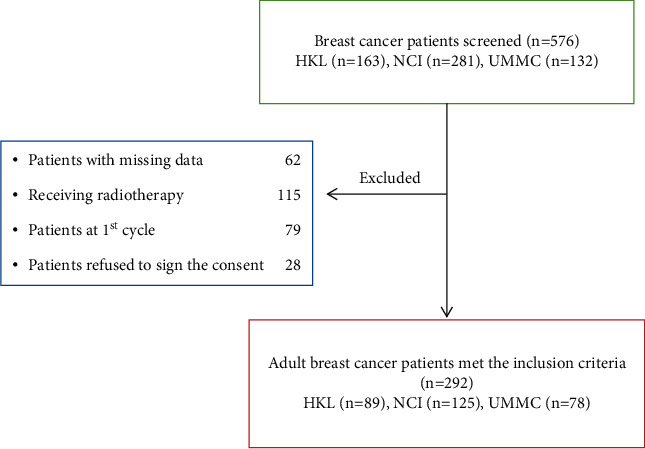
Adult patients with breast cancer screened during study period.

During the study period, 576 patients were screened at the three hospitals distributed as following: HKL (*n* = 163), NCI (*n* = 281), and UMMC (*n* = 132). One hundred and fifteen (*n* = 115) of these patients were excluded due to radiotherapy and hormonal therapy. In addition, 141 participants were dropped from the study due to missing data or because they were at the first chemotherapy cycle. Moreover, 28 patients were excluded from the study because they refused to sign the consent form. Finally, there were a total of 292 patients, HKL (*n* = 89), NCI (*n* = 125), and UMMC (*n* = 78), who were eligible for inclusion in this study (Figure 1).

### 2.7. Ethics Approval

All aspects and protocols of this study were reviewed and approved by the Research Ethics Committee of Universiti Teknologi MARA (UiTM) (REC/392/19), Clinical Research Centre (CRC) of Hospital Kuala Lumpur (HKL) (HCRC.IIR-2019-07–163), Institut Kanser Negara (IKN) (IKN/500–5/1/25 JId 4 [18], University of Malaya Medical Centre (UMMC), and the Medical Research Ethical Centre (MREC) (NMRR -18-3902-45218). The researcher followed the principles of the Helsinki Declaration and Malaysian Good Clinical Practice Guidelines.

## 3. Results

Table 1 displays clinical and demographic data for the 292 patients who participated in this study. As shown, the prevalence of anemia was 41.1% (120/292). Of the total number of patients enrolled in the study, 56.6% (68/120) had mild anemia, 34.2% (41/120) had moderate anemia, and 9.1% (11/120) had severe anemia. The average hemoglobin level was 10.34 g/dL. Furthermore, Table 1 shows that approximately 57.9% (169/292) of the patients were Malay, followed by 24% (70/292) of Chinese and 13.4% (39/292) of Indians. The average mean of hemoglobin level was 10.34 g/dL. A majority of patients were older than 60 years old (74.2%), and the average mean of age were 52.77 years. The majority of patients (74.2%) were over 60 years old, with an average age of 52.77 years. In terms of marital status, 89.4% (261/292) of the patients were married, 5.8% (17/292) were single, and 4.8% (14/292) were divorced. The majority of the patients, 59.6% (174/292), were obese, whereas 40.4% (118/292) were not. Moreover, approximately 66.8% (195/292) and 68.5% (200/292) of total participants were working and postmenopausal patients, respectively.

In this study, 42.5% (124/292) of the patients were diagnosed with Stage III followed by Stage IV, 22.3% (65/292), and Stage I, 7.5% (22/292). Additionally, nearly half of patients prescribed a single chemotherapy regimen 48.3% (141/292), and multiple chemotherapy regimens 51.7% (151/292). Finally, in terms of chemotherapy type, approximately 44.9% (131/292) received 5-fluorouracil, epirubicin, and cyclophosphamide (FEC) and 27.1% (79/292) received Docetaxel chemotherapy (Table 1).

### 3.1. Anemia Data

As shown in Table 2, the prevalence of anemia was 41.1% (120/292). Of the total number of patients enrolled in the study, 56.6% (68/120) had mild anemia, 34.2% (41/120) had moderate anemia, and 9.1% (11/120) had severe anemia. The average mean of hemoglobin level at baseline was 11.12 g/dL (SD 0.641) and declined after receiving chemotherapy to 10.34 g/dL (SD 0.728). Furthermore, anemia was classified based on MCV and MCHC values as microcytic hypochromic anemia (75% (91/120)), normocytic normochromic anemia (17.5% (21/120)), and macrocytic polychromic anemia (6.6% (8/120)). Moreover, Table 2 indicated that the majority of patients diagnosed with iron deficiency anemia (75.8% (91/120)), 16.7% (20/120) of vitamin B12 and folic acid deficiency, and 7.5% (9/120) of blood loss. Finally, the majority of anemic patients did not receive any type of anemia treatment and only 32.5% (39/120) received anemia management. Out of the 39 patients who received anemia treatment, approximately 71.8% (28/39) treated with iron therapy, folic acid, vitamin B12, and multivitamins, whereas 28.2% (11/39) underwent blood transfusion and none of the patients treated with erythropoietin-stimulating agents (ESAs) (Table 2).

### 3.2. Prevalence of Anemia among Breast Cancer Patients

The prevalence of anemia among breast cancer patients was 41.1%. Approximately 66.9% (*n* = 89) of anemic patients aged less than 60, and nearly 57.1% (*n* = 100) of patients with anemia were obese. Fifty-six (60.9%) of the prevalence of anemia was observed among premenopausal breast cancer patients. Considering the patient's cancer stage, approximately 50% (*n* = 62) of anemic patients were diagnosed with Stage III followed by Stage IV (36.9% (*n* = 24)) and Stage II (29 (35.8%)). With regard to the chemotherapy data, approximately 17.9% (*n* = 27) of patients receiving more than one chemotherapy regimen were affected with anemia and 47% (*n* = 45) prescribed docetaxel (Table 2).

### 3.3. Factors Associated with Severity of Anemia

Our data indicated that patients with anemia were older than patients without anemia (65.3 vs 54.7 years) (*p* < 0.05). In comparison with patients without anemia, a significantly greater proportion of breast cancer patients were admitted to oncology daycare units with anemia (66.9% vs 19.5%), and this association persisted after age adjustment (*p* < 0.05) (Table 2).

Furthermore, the chi-squared analysis revealed that obese breast cancer patients had a significantly higher incidence of anemia. In addition, obese patients admitted to oncology departments had a higher proportion of anemia than nonobese patients (57.5 and 42.5% vs 16.9 and 83.1%, respectively) (*p* < 0.05). In summary, based on results from logistic regression, obese patients were 12.7 times more likely to develop anemia than nonobese patients (*p* < 0.05) (Table 3).

In terms of cancer stage, of the 292 patients diagnosed with breast cancer, nearly half (50% [62/292]) of all anemic and nonanemic patients were at Stage III. The proportion of anemic patients increased with advanced stage: 36.9% (24/292) for anemic patients and 63.1% (41/292) for nonanemic breast cancer patients (Table 2).

We classified patients into groups based on the number of chemotherapy regimens they received and compared the incidence of anemia by the number of regimens. According to the chi-squared results, the highest proportions of patients prescribed with one chemotherapy were those who suffered from anemia (66% (93/292)) and only 48% (34/292) were not suffering from anemia when compared with nonanemic patients who received more than one chemotherapy regimen (17.9% (27/292) and 82.1% (124/292)) (Table 2). In comparison with patients without anemia, logistic regression analysis indicated that patients who received combination of chemotherapy were 74 times more likely to develop anemia compared to those who received only one chemotherapy regimen (*p* < 0.05) (Table 3).

Our data also showed that patients who prescribed with docetaxel chemotherapy treatment but had anemia were more likely to induce anemia than those who did not have anemia (*p* < 0.05) (Table 3).

Our findings revealed that anemia incidence was significantly associated with both clinical and demographic covariates. However, as shown in Table 4, we found that the clinical covariates explained more of the variance in the model than the demographic covariates (3 clinical variables and 2 demographic variables).

## 4. Discussion

This study successfully identified clinical and demographic characteristics of breast cancer patients that are associated with anemia prevalence. We found that determinants such as the number of chemotherapy regimens used, dose reduction, and docetaxel were independent clinical prognostic indicators of anemia prevalence. Age and BMI were the independent demographic determinants.

Anemia is a frequent complication in critically ill and cancer patients. It has been linked to a reduction in quality of life as well as a poor prognosis [4]. In addition, sadness, frustration, and exhaustion can all contribute to a patient's feeling that life is pointless because of the lowered quality of life associated with anemia [23]. Additional research has revealed an increased risk of mortality and complications such as pulmonary edema as a result of cancer-related anemia [24, 25]. Thus, identifying risk factors for anemia early may assist doctors in optimizing care for critically ill cancer patients [26].

Our data identified a high prevalence of anemia in patients with breast cancer admitted to the oncology departments and daycare centers of HKL, UMMC, and the NCI. Anemia was prevalent in 42.8% of patients at NCI, 30.5% at HKL, and 26.7% at UMMC.

Numerous researchers have previously reported a high prevalence of anemia in cancer patients in which their results were identical to the current study. For example, the ECAS [8] found that the incidence of anemia in cancer patients was 39.3%, which is slightly lower than our finding. On the other hand, our results were lower than the study conducted by Chaumard N et al. [27], who stated that 64.8% of breast cancer patients developed anemia. This proportion varied between 11 and 88% in more detailed and specific research examining adjuvant therapy [28]. We think that the difference is due to the different toxicity grading systems used in these studies: anemia was characterized as any recorded Hg level lower than 11 g/dl [28] or 10 g/dl [27], whereas the current study used a cutoff value of 12 g/dl in accordance with the National Cancer Institute grading system. In addition, patients with cancer who are also anemic may require a different clinical management strategy for their anemia in order to improve their outcomes. Furthermore, the disparity in prevalence rates may be explained by clinical features of immunocompromised patients, such as bad performance status, acute physiologic changes, and inadequate nutrition due to the severity of illness, all of which can result in cancer-related anemia [8], particularly in those patients who have undergone more aggressive treatment.

Moreover, our findings indicated that obesity increases the risk of anemia in cancer patients. According to the chi-squared analysis, 57.5% of obese breast cancer patients develop anemia, whereas only 16.9% of nonobese patients do. Logistic regression analysis confirmed these findings, revealing that obese patients were 12.4 times more likely to develop anemia than nonobese breast cancer patients. Our findings are consistent with those of Aigner E et al. [29] and Camaschella C [30], who established a statistically significant association between obesity and anemia. The suggested explanation could be that obesity impairs nutritional absorption from the duodenum, resulting in iron deficiency anemia [29]. Also, heavy eating stimulates the immune system to release excessive amounts of pro-inflammatory cytokines via iron sequestration and suppresses bone marrow's ability to produce RBCs [31]. The body cannot use stored Fe because of inflammation, which also causes anemia.

We found that breast cancer patients who did not have their chemotherapy dose reduced were 1.4 times less likely to develop anemia than those who did have a detected reduced dose (Table 3). In Table 2, the chi-squared analysis revealed a significant relationship between chemotherapy dose reduction and prevalence of anemia (*p* < 0.05). Approximately 25% of the anemic breast cancer patients had their dose reduced (Table 2). This result is slightly lower than those reported in two recent studies, which indicated that 26% [20] and 48.2% [21] of all breast cancer patients with a dose reduction. Aside from that, our finding was greater than one study's finding who reported that 8% of cancer patients had their dose reduced [32]. This discrepancy could be explained by the fact that we defined chemotherapy dose reductions as at least a 10% reduction in dose from the first follow-up [21, 22], whereas Nagel C.I et al. [32] defined them as dose modification from the first follow-up. Chemotherapy dose reductions occur most frequently during late cycles, particularly with FEC and docetaxel regimens [33]. There are several possible explanations for this decrease. First, the doses of docetaxel and FEC may be higher than those required for each regimen's efficacy, particularly in late cycles, resulting in severe toxicities that allowed the oncologist to decrease the chemotherapy dose. Second, most chemotherapy-sensitive cancer cells may be killed during the initial cycles, and then, the dose may be reduced in late cycles to avoid or minimize any negative impact of the chemotherapy. There may be a decrease in the dosage of chemotherapy as a result of the increased incidence of severity of anemia or some adverse effects of chemotherapy, such as neutropenia and neuropathy [20, 33].

The multitude of chemotherapy regimens used was found to be a clinical prognostic factor for anemia. This was expected, given chemotherapy's well-documented bone marrow depressant and acute cytotoxic properties [34]. Chemotherapy was associated significantly with anemia prevalence, and the interactions we found point to the two subgroups of patients vulnerable to anemia: the elderly and obese breast cancer patients.

In addition, our findings revealed a link between the type of chemotherapy used, such as docetaxel, and the prevalence of anemia. According to a logistic regression analysis, patients who received docetaxel developed anemia at a higher rate than those who did not receive docetaxel (Table 3). Correspondingly, similar findings indicated a significant relationship between docetaxel and anemia [35]. Despite the fact that anthracycline and taxane-based chemotherapeutic regimens have become the standard of care for breast cancer treatment, they have been shown to reduce mortality and improve survival rates. Docetaxel, on the other hand, has a number of side effects, including cardiac complications and anemia, which increases the prevalence of anemia in patients receiving these types of regimens [36]. Furthermore, the mechanism of action of these regimens may result in bone myelosuppression, which causes bone marrow damage and impairs its ability to produce enough red blood cells, white blood cells, and platelets [37].

Cancer-related anemia (CRA) develops in part as a physiological response of the immune system to the action of many cytokines on various iron homeostasis and erythrocyte production pathways, which may result in bone marrow suppression [38]. Anemia in hematologic cancer patients may be caused by the direct and indirect hematopoietic effects of uncontrolled leukocytosis and cytokine production [29]. This would explain why the incidence of anemia is higher in elderly patients admitted to oncology hospitals in this study. Although elderly cancer patients may be given less aggressive treatments than younger patients, more research is needed to better understand the determinants of incident anemia in elderly cancer patients requiring critical care.

There are numerous factors that contribute to the occurrence of anemia in cancer patients. Higher levels of inflammatory markers, interleukin-6, and leptin; hepcidin levels; ferritin levels; EPO levels; and reactive oxygen species (ROS) all contribute to the incidence of anemia in cancer patients [13]. Anemia is caused by pro-inflammatory cytokines, which directly inhibit the production of red blood cells from the bone marrow [39, 40]. Other factors such as poor nutrition, folate deficiency, lengthy hospitalization, and bleeding history all contributed to anemia incidence in cancer patients [1, 41, 42].

Our study has limitations due to a lack of information regarding anemia treatment. Erythropoietin-stimulating agents (ESAs), for example, were not administered to all patients, and only a few patients received blood transfusions. Considering that some patients received treatment for anemia, such as iron therapy, we detected a rather high prevalence of anemia in our study sample. We understand that anemia can be caused by a number of factors, including underlying cause, cancer relapse or exacerbation, infection, or renal failure.

Our study recommends all oncologists and healthcare professionals to follow the protocol of NCCN Clinical Practice Guidelines in Oncology and ESMO guidelines, which both recommend to treat cancer-related anemia based on the etiological causes and severity of anemia. If anemia is severe (Hb < 7–8 g/dL), it should be treated with blood transfusion; if anemia is moderate (Hb 8–10 g/dL), it should be treated with ESAs; and if anemia is mild (Hb 10–12 g/dL), it should be treated with iron therapy or multivitamins. ESAs should be added when necessary particularly if anemia is symptomatic and iv iron therapy is added only to improve the efficacy of ESAs and to reduce the need for red blood cell transfusion [43, 44].

## 5. Conclusion

Anemia is a common complication in breast cancer patients. The clinical and demographic factors associated with the prevalence of cancer-related anemia (CRA) in breast cancer patients were investigated in this study. The current CRA treatment guidelines should be improved to identify which patient subgroups are most likely to develop anemia. Some research is needed to determine the best way to treat anemia in cancer patients and whether or not it affects the patient's health. Our study contributes to that effort by providing data that can be used to identify groups of patients who are more likely to develop anemia. For these groups, optimal cancer management should include effective anemia treatment.

## Figures and Tables

**Table 1:** Demographic and clinical data for the 292 patients included in the study.

Variable		*N* (%)
Hospital name	HKL	93 (31.8%)
	UMMC	80 (27.4%)
	NCI	119 (40.1%)
Mean age/years	52.77 (SD 10.25)	

Age/years	≥60	159 (54.5%)
	<60	133 (45.5%)

Race	Malay	169 (57.9%)
	Indian	39 (13.4%)
	Chinese	70 (24%)
	Others	14 (4.8%)

Marital status	Married	261 (89.4%)
	Single	17 (5.8%)
	Divorced	14 (4.8%)

Body mass index (BMI) kg/m^2^	Obese BM (≥25)	118 (40.4%)
	Nonobese BMI (<25)	174 (59.6%)

Employment status	Employed	195 (66.8%)
	Nonemployed	97 (33.2%)

Social status	Smoking	20 (6.8%)
	Nonsmoking	272 (93.2%)

Menopausal status	Pre	92 (31.5%)
	Post	200 (68.5%)

Stage of breast cancer	Stage I	22 (7.5%)
	Stage II	81 (27.7%)
	Stage III	124 (42.5%)
	Stage IV	65 (22.3%)

Number of regimen	1	141 (48.3%)
	> 1	151 (51.7%)

Dose delay	Delayed	128 (43.8%)
	Not delayed	164 (56.2%)

Dose reduction	Reduced	51 (17.5%)
	Not reduced	204 (69.9%)
	Not detected	37 (12.7%)
FEC^*∗*^	Received	131 (44.9%)
Docetaxel	Received	79 (27.1%)

^*∗*^Fluorouracil, Epirubicin, Cyclophosphamide.

**Table 2:** Data of anemia among breast cancer patients receiving chemotherapy (*n* = 120).

Variable					N (%)
Prevalence of anemia	Nonanemic				172 (58.9%)
	Anemic				120 (41.1%)
Severity of anemia *n* = 120	Mild				68 (23.2%)
	Moderate				41 (14%)
	Severe				11 (3.7%)
Hb level mean at baseline (g/dL)	11.12 g/dL (SD 0.641)				
Hb level mean after receiving chemotherapy (g/dL)^a^	10.34 g/dL (SD 0.728)				
Anemia types *n* = 120	**<** 80	Microcytic	**<** 32	Hypochromic	91 (75.8%)
	80–100	Normocytic	32–36	Normochromic	21 (17.5%)
	**>** 100	Macrocytic	**>** 36	Polychromic	8 (6.6%)
Classification of anemia ^*∗*^*n* = 120	Iron deficiency anemia				91 (75.8%)
	Vitamin B12 and folic acid deficiency				20 (16.7%)
	Blood loss				9 (7.5%)
Treatment of anemia *n* = 39	Iron therapy				11 (28.2%)
	Vitamin B12				6 (15.4%)
	Vitamin C				6 (15.4%)
	Multivitamins				5 (12.8%)
	Erythropoietin-stimulating agents (ESAs)				
	Blood transfusion				11 (28.2%)

^a^The mean of Hb level is the average mean for the three follow-ups. ^*∗*^Classification of anemia based on MCV and MCHC [23].

**Table 3:** Association between prevalence of anemia and demographic and clinical factors.

Variable		Anemia		*p*–value^a^
		With Frequency of Anemia, *n* (%)	Without Frequency of Anemia, *n* (%)	
Age (years)	**< 60**	31 (19.5%)	128 (80.5%)	0.001
	**≥ 60**	89 (66.9%)	44 (33.1%)	
BMI kg/m^2^	Obese (≥25)	100 (57.5%)	74 (42.5%)	0.002
	Nonobese (<25)	20 (16.9%)	98 (83.1)	
Menopausal status	Pre	56 (60.9%)	36 (39.1%)	0.001^*∗*^
	Post	64 (32%)	136 (68%)	
Cancer stage	Stage I	5 (22.7%)	17 (77.3%)	0.036^*∗*^
	Stage II	29 (35.8%)	52 (54.2%)	
	Stage III	62 (50%)	62 (50%)	
	Stage IV	24 (36.9%)	41 (63.1%)	
Number of regimens	**1**	93 (66%)	48 (34%)	0.001
	> **1**	27 (17.9%)	124 (82.1%)	
Dose reduced	Reduced	23 (45.1%)	28 (54.9%)	0.001
	Not reduced	66 (32.8%)	138 (67.2%)	
	Not detected	31 (83.8%)	6 (16.2%)	
Dose delay	Delayed	62 (48.4%)	66 (51.6%)	0.024
	Not delayed	58 (35.4%)	106 (64.6%)	
Docetaxel	Received	45 (47%)	34 (43%)	0.001
	Not received	75 (35.2%)	138 (64.8%)	

^a^Chi-squared analysis. *p* < 0.05 indicates a level of significance. ^*∗*^Fisher exact test. *p* < 0.05 indicates a level of significance.

**Table 4:** Type of relationship between prevalence of anemia and demographic and clinical factors.

Variables	Prevalence of Anemia				
	b	OR	CI (95%)		*p* Value^a^
			Lower	Upper	
Age group					
The Elderly ≤60 years	Reference				
Young age **>** 60 years	2.015	7.5	3.062	18.388	≤0.001
BMI kg/m^2^					
Not obese **(**≤25)	Reference				
Obese (>25)	2.5	12.4	4.55	34.07	≤0.001
Number of regimens					
1	Reference				
**>** 1	4.305	74.08	21.9	249.7	≤0.001
Dose reduction					
Not detected	Reference				
Reduced	0.414	1.514	1.514	.518	0.448
Not reduced	- 4.961	1.419	.007	.001	≤0.001
Chemotherapy type					
Docetaxel received	Reference				
Not received	-1.381	0.251	.092	.687	0.007

^a^Logistic regression analysis. *p* < 0.05 indicates a level of significance. ^a^FEC, fluorouracil, epirubicin, cyclophosphamide.

## Data Availability

The prospective data used to support the findings of this study are available from the author (Fares M.S Muthanna) upon request.
